# Mediation role of body fat distribution (FD) on the relationship between *CAV1 rs3807992* polymorphism and metabolic syndrome in overweight and obese women

**DOI:** 10.1186/s12920-021-01050-6

**Published:** 2021-08-12

**Authors:** Faezeh Abaj, Said Abdul Ghafour Saeedy, Khadijeh Mirzaei

**Affiliations:** 1 Department of Community Nutrition, School of Nutritional Sciences and Dietetics, Tehran University of Medical Sciences (TUMS), P.O. Box 14155-6117, Tehran, Iran; 2 Department of Paraclinic, School of Medicine, Herat University, Herat, Afghanistan

**Keywords:** Caveolin-1, Polymorphism, Metabolic syndrome, Indirect effect, Body fat distribution

## Abstract

**Background:**

Metabolic syndrome (MetS) is associated with an increased risk of morbidity and mortality in almost all chronic diseases. The most frequent methods for the calculation of a continuous MetS (cMetS) score have used the standardized residuals in linear regression (z-score). Recently, emerging data suggest that one of the main genetic targets is the *CAV1*, which plays a crucial role in regulating body fat distribution. This study is designed to investigate the relationship between *CAV1 rs3807992* genotypes and cMetS, and to determine whether body fat distribution plays a mediating role in this regard.

**Methods:**

The current cross-sectional study was conducted on 386 overweight and obese females. The *CAV1 rs3807992* and body composition were measured by the PCR–RFLP method and bioelectrical impedance analysis, respectively. Serum profile of HDL-C, TGs, FPG, and Insulin were measured by standard protocols.

**Results:**

GG allele carriers had significantly lowered Z-MAP (*p* = 0.02), total cMetS (*p* = 0.03) and higher Z-HDL (*p* = 0.001) compared with (A) allele carriers. There was a significant specific indirect effect (standardized coefficient = 0.19; 95% CI 0.01–0.4) of Visceral fat level (VFL). Although, total body fat was significantly associated with *CAV1 rs3807992* and cMetS, the specific indirect effect was not significant (standardized coefficient = 0.21; 95% CI − 0.006, 0.44). VFL contributed to significant indirect effects of 35% on the relationship between *CAV1* and cMetS.

**Conclusion:**

Higher visceral adipose tissue may affect the relationship between *CAV1* and cMetS. Although *CAV1 rs3807992* is linked to VFL in our study, the influence of this polymorphism on MetS is not via total fat.

## Background

MetS refers to a set of metabolic disorders that are associated with a high risk of cardiovascular disease. Its components include abdominal obesity, high blood pressure (BP), hypertriglyceridemia, hyperglycemia, and low level of high-density lipoprotein-cholesterol (HDL-C) [1, 2]. Based on large investigations, MetS is associated with an increased risk of morbidity and mortality in almost all chronic diseases [1, 3]. There is lack of consensus on a precise definition for MetS. This has brought about several definitions and strategies for this condition [4]. During the last decade, symmetrical to the changes in definitions of the MetS, an alternative approach for the monitoring of MetS was also improved. Given the lack of considerable evidence for using a dichotomous MetS definition because of information loss and the absence of a universal definition for MetS, a potential way for using a continuous score to quantify the MetS was developed [5].

So far, the most frequent methods for the calculation of a cMetS score have used the standardized residuals in linear regression (z-score) [6]. The MetS severity z-score, an inexpensive and clinically-available continuous measure of MetS, was derived from standardized loading coefficients for fasting blood sugar (FBS), triglycerides (TGs), waist circumference (WC), BP, and HDL-cholesterol [7].

Amazingly, evidence suggests that genetic variations, in addition to obesity, play a major role in regulating body fat composition. [8]. It appears that genetic factors have a more prominent impact on visceral fat than on other adipose tissues. It is suggested by some authors that apart from TBF, genetic factors have a great effect on visceral fat [9–11]. The availability of enormous genomic information, such as Genome-Wide Association Studies (GWAS), has facilitated the field for fine-mapping and translation of genetic information into clinical practice [12]. Recently, emerging data suggest that one of the main genetic targets is the *CAV1*, which plays a crucial role in regulating FD [13, 14]. *CAV1* is the main part of caveolae and has been extensively studied in dyslipidemia and cardiovascular diseases for its important role in the signal transduction, interaction with steroid receptors, involvement in the activation of ion channels, and cholesterol hemostasis [15, 16].

In this research, we focused on the *CAV1* gene which is located on chromosome 7q31.2. *CAV1* [15]. *CAV1* was found to be expressed at a high level in adipocytes. Moreover, *CAV1* is involved in adipogenesis pathway as well as lipogenesis [17]. It is shown by a previous study that a significant association exists between higher *CAV1* expression in visceral adipose tissue (VAT) and in subcutaneous adipose tissue (SAT) of obese patients [18].

Numerous gene loci that have regulator action on the body FD are revealed by recent GWAS for a measure of FD [10]. The sexual dimorphic effect has been revealed by sex-stratified studies at twenty Waist-Hip Ratio (WHR)-associated loci. Nineteen out of these twenty loci exhibited more powerful effects in women [19, 20]. Lack of caveolae, as seen in lipoatrophic caveolin-deficient patients, severely compromises fat cell expansion [21]. The risk of developing obesity-related metabolic and cardiovascular complications such as MetS is affected in large quantities by adipose tissue dysfunction and ectopic fat [22].

*CAV1* is now specified as a gene involved in FD and potential mechanisms contribute to its variability between VFL and TBF. Regarding this, we have investigated the effect of these pathways and their association with *CAV1* and MetS.

The main purpose of this study is to assess the relationship between *CAV1 rs3807992* genotype*s* and the risk of MetS and to determine whether body FD plays a mediating role in this regard.

## Methods

### Samples

A total of 386 overweight and obese women within the range of 18–55 years old were included in the study [23]. The exclusion criteria were cardiovascular diseases (CVDs), kidney failure, stroke, thyroid disease, liver disease, cancer, inflammatory illnesses, pregnancy, and any therapeutic medications. Each participant provided written informed consent. The study was approved by the Ethics Community of Tehran University of Medical Sciences (TUMS) (97-03-161-41017).

### Anthropometric assessments and body composition

Bodyweight and height were measured when the subjects were minimally clothed and not wearing shoes in a standing position using a digital scale (Seca, Germany) and tape measure with a precision of 100 g and 0.1 cm, respectively. WC was measured at the narrowest area below the rib cage and above the umbilicus using a non-elastic tape to the nearest 0.1 cm. Body Mass Index (BMI) was calculated as weight (kg) divided by square of height (m^2^). A validated International Physical Activity Questionnaire was used for the assessment of the physical activity [24]. After that, for the measurement of BP, participants sat in a rest position for 10 min. They were in suitable positioning including back support. Using a properly sized cuff, their BP was measured from the left bare arm which was supported at heart level [25].

BMI values between 25 and 29.9 kg/m^2^ and above 30 kg/m^2^, were defined as overweight and obese, respectively. VFL, TBF, percent body fat (%BF), skeletal muscle mass (SMM), soft lean mass (SLM), fat mass index (FMI), and obesity degree were measured by tetrapolar bioelectrical impedance analysis (InBody 770 scanner, Seoul, Korea), according to the manufacturer's guidelines [26]. First, participants removed their shoes, coats, and sweaters; then they stood barefoot on the balance scale and held the handles of the machine.

### Clinical analysis and DNA genotyping

All serum samples were collected after overnight fasting (12-14 h) at the Nutrition and Genomics Laboratory of TUMS [27]. We stored plasma samples at − 80 °C. Baseline circulating fasting blood sugar (FBS), plasma lipid (TGs), and lipoprotein (HDL) were profiled by a standard enzymatic technique with a commercial kit (Pars Azmoon Co., Tehran, Iran). DNA extraction was accomplished from the whole blood sample by a Mini Columns kit (Type G; Genall; Exgene). PCR–RFLP method was used to determine *CAV1 rs3807992* polymorphism. For amplification, we utilized the following primers: F:5′AGTATTGACCTGATTTGCCATG-3′, R:3′-GTCTTCTGGAAAAAGCACATGA-5′. Finally, the PCR was conducted in a final volume of.

20 µl, containing 1 µl extracted DNA, 1 µl Forward primers, 1 µl Reverse primers, 7 µl distilled water, and 10 µl Taq DNA Polymerase Master Mix (Ampliqon; Germany) under the following conditions: each reaction started with a cycle of DNA templates denatured at 94 °C for three minutes. Amplification consisted of forty cycles (each cycle includes 94 °C for fifteen seconds, 53 °C for thirty seconds, 72 °C for thirty seconds), with a final extension at 72 °C for three minutes. 10 µl of DNA was digested with 0.5 µl of the Hin1II(NlaIII) enzyme (Fermentase, Germany) at 37 °C overnight. Electrophoresis was conducted to visualize all PCR products. We employed TRIS–Borate-EDTA buffer (TBE) which was prepared as a 10X concentrated stock. PCR–RFLP products were detected by 3.5% gel electrophoresis**.** The gels were visualized by UV light by two independent individuals.

### Calculation of a cMetS score

To derive cMetS, regression analysis of standardized residuals of the WC, FBS, HDL-C, TGs, and mean arterial pressure (MAP) was done on age to adjust them for age-related differences. Since the standardized HDL-C is conversely associated with the MetS’s risk, it was multiplied by (− 1). MAP calculated from measured systolic blood pressure (SBP) and diastolic blood pressure (DBP) variables. MAP was calculated by this formula: [(SBP − DBP)/3] + DBP. The sum of the standardized residuals (z-score) for the subjects’ variables was used to compute a cMetS score.

### Statistical methods

The Kolmogorov–Smirnov was used to test normality before computing the z-score. Eighteen of the original 404 participants were identified as outliers and were removed, so that the final analysis was done on 386 participants.

The Exact test was used for the Hardy–Weinberg Equilibrium (HWE). One-Way ANOVA and ANCOVA tests were used for determining the association between quantitative variables. These variables include Age (year), BMI (kg/m^2^), WC (Cm), WHR, BFM (kg), FFM (kg), SMM (kg), SLM (kg), BF%, VFL, VFA (cm^2^), Obesity degree %, FFMI (kg), FMI (kg), Total body mineral content (kg). These variables were organized in three groups of genotypes in the crude and adjusted model, respectively. Moreover, the independent-sample t-test was used to compare the above continuous variables according to two groups of an allele in the dominant model. Findings on continuous variables were expressed as means ± standard deviation (SD). *p* < 0.05 was considered statistically significant.

To investigate the mediatory role of body FD in the relationships between *CAV1* polymorphism and cMetS, we used tests of mediation which is an important, new statistical technique in nutrigenetic science to test hypothesized processes (such as body distribution), predictor variable (such as genetic susceptibility) and dependent outcome (such as MetS).

Mediation analysis assesses whether supposed mediating variables explain the relationship between a predictor variable and the outcome variable. For the first time, Baron and Kenny developed this analysis [20]. This method uses bootstrapping to make 95% confidence intervals to detect the indirect effect. If zero is not within this confidence interval, the indirect effect is significant.

**Fig. 1:**
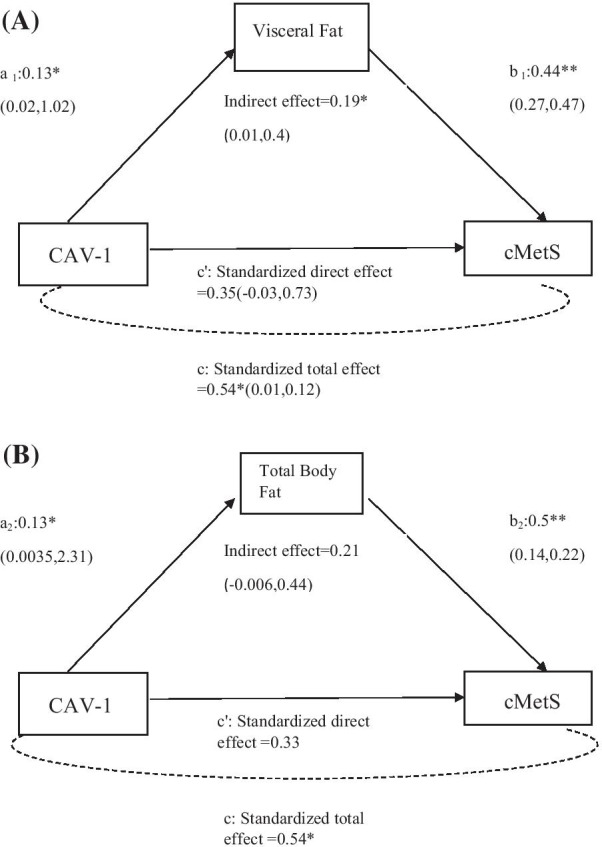
Mediation effects of VFL and TBF on the association between Caveolin-1 rs807992 polymorphism and cMetS (VFL; **A**), (TBF; **B**). Confounding factors were age, energy intake. Standardized coefficients were shown along with their estimated *p* values: “a” is the linear regression coefficient of the CAV-1rs3807992- cMetS association; “b” is the linear regression coefficient of the VFL and TBF of cMetS. *p < 0.05; ** *p* < 0.001. CAV-1 coded for the number of risk alleles present carried by an individual (GG = 0 AG + AA = 1). The analysis was done using SPSS Process Andrew 3.3 with 5000 bootstrap samples. Statistically significant paths do NOT contain zero between lower and upper level confidence intervals

As shown in Fig. 1, path c refers to the total effect of *CAV1* on cMetS in the absence of the mediators. Specific indirect effects refer to the influence of (path a × path b) via each particular mediator, (a1 × b1) is the indirect effect of *CAV1* on cMetS by VFL; (a2 × b2) is the indirect effect of *CAV1* on cMetS by TBF. Path c’ refers to the direct effect of *CAV1* on cMetS when also controlling for the mediators.

A test of significance of indirect effects was performed by Bias-corrected bootstrap confidence intervals (n = 5000, confidence intervals set at 95%), which were established by Hayes [28].We used SPSS 25 for all statistical analyses.

## Results

386 overweight and obese women were investigated in this cross-sectional study. The means and standard deviation (SD) of age, BMI, and WC of individuals were 36.67 ± 9.1 years, 31.26 ± 4.29 kg/m^2^, and 99.61 ± 10.07 cm, respectively. The overall prevalence of *rs3807992* genotypes was 38.34% and 61.66% for the A, and G alleles. The genotype distributions were within HWE (*p* value > 0.01). Based on *CAV1 rs3807992*, we had three groups of participants (AA, AG, and GG). The information of their characteristics is shown in Table 1. The mean FMI, VFL, and obesity degree (%) were significantly higher in AA compared to AG and GG (*p* < 0.05). Also, we couldn’t find any other significant difference among *CAV1* rs3807992 groups. Besides, in dominant comparison, the results of the study revealed that mean BFM, VFL, and FMI were significantly lower in GG homozygotes genotypes, compared with A-allele carriers (*p* < 0.05).

**Table 1:** Baseline characteristics of all participants according to genotype CAV-1 rs3807992

Variables	AA (n = 103)	AG (n = 90)	GG (n = 193)	*p* value	(AA + AG)/GG	*p* value
Age(year)	35.67 ± 8.71	*35.85* ± 8.91	37.56 ± 9.49	0.15	35.75 ± 8.78	0.05
BMI (kg/m^2^)	31.64 ± 3.96	30.93 ± 3.89	30.57 ± 3.88	0.09	31.30 ± 3.93	0.07
WC (cm)	100.52 ± 9.38	99.13 ± 9.65	98.22 ± 9.3	0.14	99.87 ± 9.49	0.08
WHR	0.94 ± 0.05	1.94 ± 9.59	0.93 ± 0.05	0.2	1.41 ± 6.58	0.31
BFM (kg)	36.51 ± 10.04	34.67 ± 7.97	33.49 ± 7.96	**0.01**	35.64 ± 9.15	**0.01**
FFM (kg)	46.80 ± 5.76	46.25 ± 5.90	46.26 ± 5.48	0.71	46.54 ± 5.83	0.62
SMM (kg)	25.68 ± 3.40	25.57 ± 3.73	25.34 ± 3.27	0.69	25.63 ± 3.55	0.4
SLM (kg)	43.66 ± 4.97	43.55 ± 5.64	43.44 ± 5.24	0.94	43.86 ± 5.53	0.44
BF%	42.86 ± 6.25	42.49 ± 4.53	41.62 ± 5.47	0.15	42.68 ± 5.5	0.05
VFL	16.42 ± 3.22	16.12 ± 2.99	15.46 ± 3.4	**0.04**	16.28 ± 3.11	**0.01**
VFA (cm^2^)	175.05 ± 40.89	165.71 ± 36.91	170.42 ± 125.32	0.78	170.65 ± 39.24	0.98
Obesity degree %	149.91 ± 22.57	142.1 ± 23.55	142.7 ± 18.96	**0.01**	146.23 ± 23.30	0.1
FFMI (kg)	18.1 ± 1.56	17.74 ± 1.66	18.42 ± 9.5	0.72	17.93 ± 1.62	0.47
FMI (kg)	14.19 ± 3.76	13.44 ± 3.06	12.93 ± 3.25	**0.01**	13.83 ± 4.61	**0.009**
Total body mineral content (kg)	2.62 ± 0.34	2.64 ± 0.34	2.65 ± 0.35	0.76	2.64 ± 0.35	0.76

Bold text indicates a statistically significant difference with a* p*-value < 0.05

BFM, Body Fat Mass; FFM, Fat Free Mass; SMM, Skeletal Muscle Mass; BF, Body Fat SLM, soft lean mass; VFA, visceral fat area; WC, waist circumference; BF, body fat; WHR, waist height ratio

Obesity Degree (%) = (current weight/standard weight by height) × 100

Pvalue = Values are means in crude model

The mean values for each part of the cMetS score which according to different *CAV1* genotypes is divided into different groups are presented in Table 2. There was a significant correlation between z-score (MAP, HDL-C) and cMetS with *CAV1* polymorphism in this study. GG group had significantly lowered (Z-MAP (*p* = 0.02), total cMetS (*p* = 0.03)) and higher Z-HDL-C (*p* = 0.001) compared with the AA and AG groups. Moreover, in the dominant model, risk allele carriers had significantly higher Z-MAP (*p* = 0.006), total cMetS (*p* = 0.01)), and lower Z-HDL (*p* < 0.001).

**Table 2:** Mean values of components of continuous metabolic risk score by CAV-1 rs3807992

	Codominant				Dominant		Recessive	
	AA	AG	GG	*p* value	(AA + AG)	*p* value (AA + AG)/GG	(AG + GG)	*p* value (AG + GG)/AA
	Mean ± SD	Mean ± SD	Mean ± SD		Mean ± SD		Mean ± SD	
Z_WC	0.1496 ± 0.994	− 0.0014 ± 1.02	− 0.1065 ± 0.979	0.11	0.07735 ± 1.01	0.07	− 0.07 ± 0.99	0.05
Z_MAP	0.1492 ± 0.891	0.1866 ± 1.19	− 0.1704 ± 0.978	**0.02**	0.1646 ± 1.02	**0.006**	− 0.07 ± 1.05	0.1
Z_FBS	− 0.0472 ± 0.86	− 0.0308 ± 1.19	0.0324 ± 0.992	0.84	− 0.0404 ± 1.008	0.56	0.01 ± 1.05	0.66
Z_HDL-C	− 0.1613 ± 0.842	− 0.3417 ± 1.05	0.2358 ± 1.02	**0.001**	− 0.2365 ± 0.93	**< 0.001**	0.07 ± 1.06	0.07
Z_Triglycerides	0.112 ± 1.19	0.1523 ± 1.05	− 0.104 ± 0.864	0.19	0.1285 ± 1.13	0.07	− 0.03 ± 0.92	0.31
Z_TOTAL	0.3897 ± 2.67	0.2624 ± 3.739	− 0.6099 ± 2.42	**0.03**	0.3390 ± 3.125	**0.01**	− 0.38 ± 2.83	0.05

Bold text indicates a statistically significant difference with a* p*-value < 0.05

Z: standardized components of a continuous metabolic syndrome risk score. WC, waist circumference; FBS, Fasting Blood Glucose; MAP, Mean Arterial pressure

Based on the recessive model, also minor allele homozygotes (AA) have a higher risk of metabolic syndrome, but this association was not significant (*p* = 0.05). Although we did not observe a significant result in the recessive model, we detected the same direct association as the dominant models. It means that we observed that AA homozygotes have a higher risk of metabolic syndrome compared to major allele carriers (AG and GG) in this model.

Mediation analysis for VFL and TBF is presented in Fig. 1. The analysis showed that the direct effect of *CAV1* polymorphism on cMetS was not significant, but only the VFL created a significant specific indirect effect (standardized coefficient = 0.19; 95% CI 0.01–0.4) after controlling for age and energy intake. Although, TBF was significantly associated with *CAV1 rs3807992* and cMetS, the specific indirect effect was not significant (standardized coefficient = 0.21; 95% CI − 0.006, 0.44). Therefore, VFL contributed to significant indirect effects of 35% on the relationship between *CAV1 rs3807992* and cMetS.

## Discussion

The current study aimed to investigate the mediation role of body FD between *CAV1* polymorphism and MetS. In the present study, we observed that risk allele carriers (AA, AG) have higher BFM, VFL, and FMI. Therefore, carriers of A allele might be at more risk of developing MetS compared to the individuals with GG genotype. Since the attribution mechanism of *CAV1* variants to MetS is unclear at the present, this association has been studied by a small number of existing researchers [29, 30] and the present study is the first human study for showing the association between *CAV1 rs3807992* polymorphism and MetS. Similar to our findings, Baudrand et al. revealed that *CAV1 rs926198* variant is associated with MetS [29]. Moreover, Mora-Garcia et al. suggest that minor alleles for SNPs *rs3779512*, *rs7804372*, and *rs1049337* might be associated with a higher risk of hypertriglyceridemia [30].

Furthermore, regardless of the relationships among the caveolae function, adipose tissue, and lipid profile [29, 31, 24], MetS which is linked to *CAV1* has not been studied regarding body FD. To a notable extent, we offered a hypothetical mechanism whereby *CAV1* can increase MetS risk via FD. As mentioned in the results section, although the *rs3807992* polymorphism was statically related with visceral and total body fat, we found evidence in mediation analysis, that only VFL explains the association between *rs3807992* and MetS. To the best of our knowledge, the indirect effect of *CAV1 rs3807992* on MetS via VFL is a new approach that has not been shown or even suggested before.

A close relationship is present between metabolic disorders and FD; the risk of developing MetS increases in people who have fat deposition in visceral adipose than TBF deposition [32]. Of particular note, the present mediation analysis is especially interesting in that the metabolic difference which is present between visceral and TBF is known [33]. The importance of visceral adipose tissue in the pathophysiology of metabolic disorders can be highlighted by comparing the specifications of VAT with SAT. VAT is linked with the development of insulin resistance, is lipolitically more active, and carries more risk factors for the development of obesity, dyslipidemia, cardiovascular and metabolic disease than subcutaneous fat [34–36]. Regarding these special characteristics, visceral fat led to many metabolic disorders such as alteration in lipid profile (low HDL-C and high TGs levels) and disturbance in glucose homeostasis [37–39]. However, several studies have suggested that these differences in FD in tissues could be genotype-related [19, 40].

According to the mentioned evidence and our pervious study [41], our results cast a new light and mechanism on the mediation role of VAT rather than TBF with consideration to *CAV1*-linked MetS. Specifically, our primary results suggest a possible pathway to MetS’s risk by VAT and the presence of the *CAV1 rs3807992* risk allele.

It is remarkable that lipodystrophies with a defective local fat deposition that are seen in mutations of *CAV1* gene indicate a new locus in the metabolic diseases [42]. It has been established by a previous study that *CAV1* has a significant regulator role at FD and genetic lipodystrophies in humans [43]. Previous findings had not indicated a special direct or indirect role for VFL in the increased development of MetS in individuals carrying the risk allele. However, studies show the association of *CAV1* mRNA expression in VAT in obese women compared with lean subjects [17, 18]. Basic studies have shown that the lean body phenotype of *CAV1*- null mice are smaller than their wild-type counterparts [36, 44]. Lipodystrophy has been caused in *CAV1* null mice due to different functions that are attributed to caveolae in adipocytes; dysfunction of the lipid droplet, disturbance in adipocyte differentiation pathway, abnormality in binding, transport, and storage of cholesterol and fatty acids, and increase in insulin signaling. These possible functional roles for *CAV1* may be inherited [43, 45].

The association of *CAV1* with body composition in our study could suggest a *CAV1*–lipogenic pathway interaction. Especially considering that *CAV1* is highly expressed in adipose tissue, and the interplay between *CAV1* gene and lipogenic genes has been reported [17]. This finding is consistent with what has been found in former studies. Excess visceral fat could be related to changes in circulating fatty acid composition [46]. Altered activities of fatty acid desaturases disturb plasma fatty acid metabolism and cause adipose tissue dysfunction. However, it seems that a possible genetic interaction between *CAV1* and fatty acid composition with the lipogenic system genes may also be accountable for our result in this study.

The present study also might have a clinical implication, highlighting the potential of applying this finding in the prevention and treatment of *CAV1*-linked obesity and cardiovascular disease that is the result of high VFL.

Since the beginning of human studies on the caveolin gene, limitations were present in those studies due to lack of information on body composition [29, 31, 47–51]. So, researchers have not been able to investigate the visceral fat factor which may have been the cause of some observed results. Therefore, with this groundbreaking work, we have researched this gap.

## Limitations

Despite our novel findings, it suffers from some limitations. All participants in our study were women, and there have been studies reporting sex differences for the effect of *CAV1* on various body compositions [17]. Other limitations of the present study include the cross-sectional design (so that any causality cannot be argued) and small sample size (which may have led to weak statistic determine significant results). Furthermore, our participants were from the country of Iran, so that the results may not be generalized due to racial and regional differences. Finally, many predisposing factors that increase with age, such as insulin resistance, inflammation and hypertension also may contribute to the increase in the prevalence of MetS (4, 5). Although in our study, the participants were within the range of 18–55 years old, we suggest that future studies be done on a wider age range.

## Conclusions

It appears that increased VAT fat accounts for the association between *CAV1 rs3807992* and MetS. Although *rs3807992* is linked to visceral fat in our study, the influence of this polymorphism on MetS is not via total fat. If replicated, this suggested pathway has the potential to have an important impact on our understanding of *CAV1*-linked MetS.

## Data Availability

The data are not publicly available due to containing private information of participants. Data are however available from the authors upon reasonable request and with permission of Khadijeh Mirzaei.

## Abbreviations

BIA: Bioelectrical impedance analysis
BMI: Body Mass Index
BP: Base pair
BFM: Body fat mass
BF: Body fat
CVDs: Cardiovascular diseases
DBP: Diastolic blood pressure
FBS: Fasting blood sugar
FD: Fat distribution
FMI: Fat Mass Index
FFMI: Fat-free Mass Index
GWAS: Genome-wide Association Studies
HDL: High-density Lipoprotein Cholesterol
IR: Insulin resistance
LDL: Low-density Lipoprotein
MA: Minor allele
MAP: Mean arterial pressure
PCR: Polymerase Chain Reaction
RFLP: Restriction Fragment Length Polymorphism
SBP: Systolic blood pressure
SNP: Single nucleotide polymorphism
SMM: Skeletal muscle mass
SLM: Soft lean mass
TG: Triglyceride
TBF: Total body fat
VAT: Visceral adipose tissue
VFL: Visceral fat level
WC: Waist circumference
WHR: Waist-Hip ratio
